# Characterization of T-Cell receptor repertoire in immunoglobulin a nephropathy

**DOI:** 10.1186/s40364-024-00572-2

**Published:** 2024-02-12

**Authors:** Szu-Ying Ho, Chih-Chin Kao, Che-Mai Chang, Yi-Chien Chou, Wei-Tzu Luo, Wan-Hsuan Chou, I-Lin Tsai, Mai-Szu Wu, Wei-Chiao Chang

**Affiliations:** 1 Department of Clinical Pharmacy, School of Pharmacy, Taipei Medical University, No. 250, Wuxing St., Xinyi District, Taipei City, 11031 Taiwan; 2 Division of Nephrology, Department of Internal Medicine, School of Medicine, College of Medicine, Taipei Medical University, No. 250, Wuxing St., Xinyi District, Taipei City, 11031 Taiwan; 3 Division of Nephrology, Department of Internal Medicine, Taipei Medical University Hospital, Taipei, Taiwan; 4 Taipei Medical University Research Center of Urology and Kidney, Taipei Medical University, Taipei, Taiwan; 5 Master Program in Clinical Genomics and Proteomics, School of Pharmacy, Taipei Medical University, Taipei, Taiwan; 6 Department of Biochemistry and Molecular Cell Biology, School of Medicine, College of Medicine, Taipei Medical University, Taipei, Taiwan; 7 Division of Nephrology, Department of Internal Medicine, Shuang Ho Hospital, Taipei Medical University, New Taipei City, Taiwan; 8 Department of Medical Education and Research, Integrative Research Center for Critical Care, Wan-Fang Hospital, Taipei Medical University, Taipei, Taiwan; 9 Department of Pharmacy, Wan Fang Hospital, Taipei Medical University, Taipei, Taiwan

**Keywords:** Immunoglobulin A nephropathy (IgAN), T-cell receptor (TCR) repertoire, TCR sequencing

## Abstract

Immunoglobulin A nephropathy (IgAN) is an autoimmune disease characterized by abnormal IgA deposition in glomerulus. Current diagnosis of IgAN still depends on renal biopsy, an invasive method that might increase the risk of clinical outcomes. Therefore, we aimed to explore the characteristics of T cell repertoire in IgAN from peripheral blood samples for identifying innovative diagnostic biomarkers. Herein, we included 8 IgAN patients, 25 non-IgAN patients, and 10 healthy controls in the study. A high-throughput immune repertoire sequencing was conducted to investigate the T-cell receptor beta-chain (TCRβ) repertoire of peripheral blood. Characteristics of TCRβ repertoire were assessed for these three distinct groups. A reduced TCRβ repertoire diversity was observed in IgAN patients compared to non-IgAN and healthy individuals. A skewed distribution toward shorter TCRβ complementarity determining region (CDR3) length was found in non-IgAN relative to IgAN patients. In addition, the differences in usages of five TRBV genes (*TRBV5-4*, *TRBV6-4*, *TRBV12-1*, *TRBV16*, and *TRBV21-1*) were identified between IgAN, non-IgAN, and healthy subjects. Of note, the *TRBV6-4* gene, which is associated with mucosal-associated invariant T (MAIT) cells, exhibited higher usage in IgAN patients, suggesting potential importance of MAIT cells in IgAN. In short, our findings supported TCR repertoire characteristics as potential biomarkers for IgAN diagnosis.

**Supplementary Information:**

The online version contains supplementary material available at 10.1186/s40364-024-00572-2.

To the editor,

Immunoglobulin A nephropathy (IgAN) is an autoimmune kidney disease caused by the deposition of abnormal immunoglobulin A (IgA) in renal tissues. It is the most common cause of primary glomerulonephritis worldwide, resulting in progression to end-stage renal disease (ESRD) in 30–40% of IgAN patients within 20–30 years [1]. Currently, renal biopsy as an invasive procedure remains the gold standard for a definitive diagnosis of IgAN [2]. There is thus an urgent need to explore novel strategies to detect IgAN.

Previous studies have highlighted the importance of T-cell profiles in IgAN [3–7]. However, more studies are still needed to determine whether TCR repertoire characteristics can be utilized as potential biomarkers for IgAN. Herein, we collected peripheral blood samples and conducted a TCR repertoire sequencing analysis to compare TCR beta-chain (TCRβ) repertoire among 8 IgAN patients, 25 non-IgAN patients (10 and 15 with diabetic nephropathy and hypertensive nephropathy, respectively), and 10 healthy individuals (Fig. 1A, Table 1, and Table S1).

**Fig. 1:**
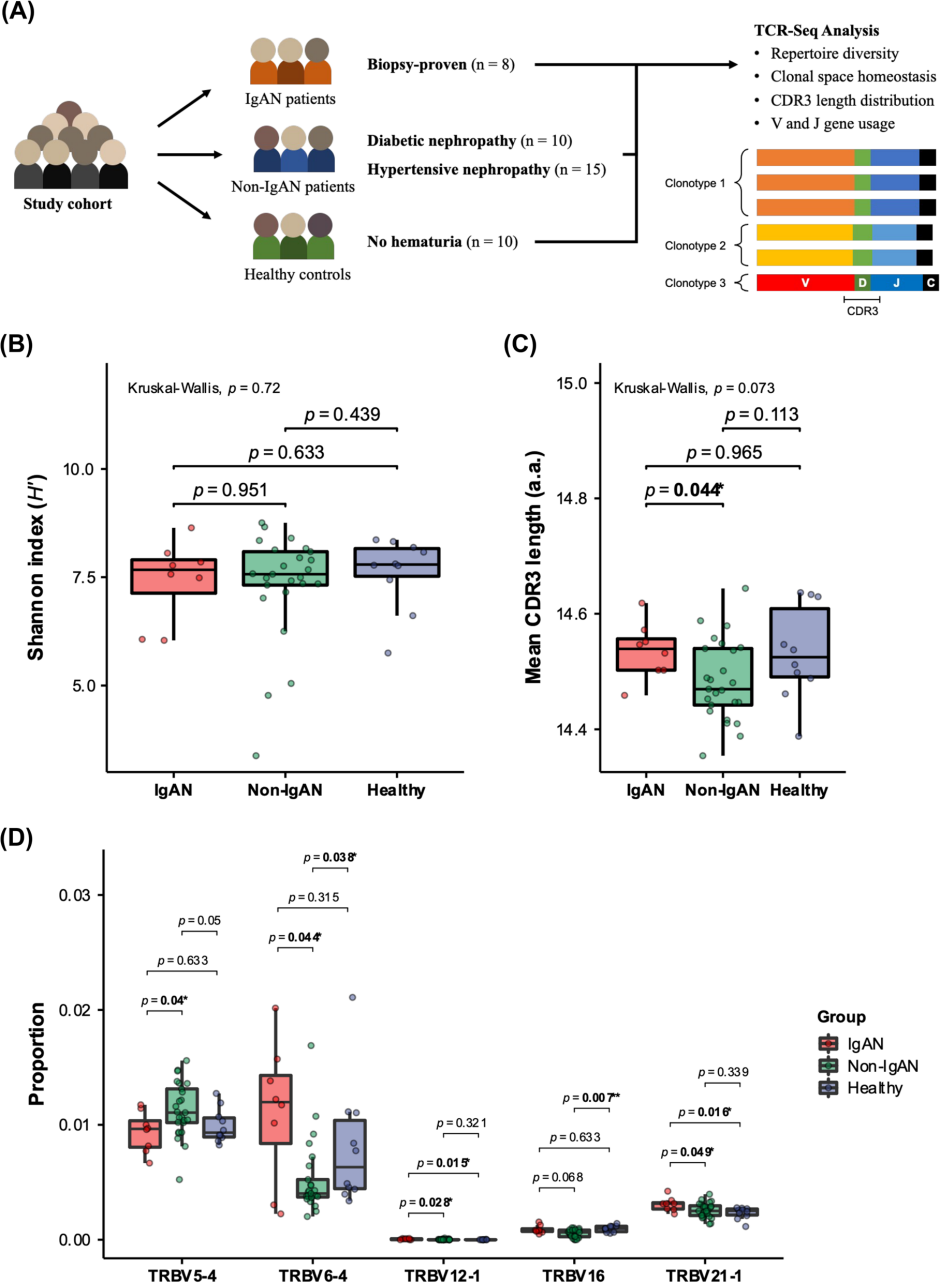
Comparison of T-cell receptor beta-chain (TCRβ) repertoire between IgAN, non-IgAN, and healthy group. **A** Flow diagram of sample collection and TCR sequencing (TCR-seq) analysis. **B** The Shannon index shown in IgAN (red), non-IgAN (green), and healthy (blue) subjects. Differences in the repertoire diversity index were calculated by the Kruskal–Wallis test (among three groups) and the Wilcoxon rank sum test (between any two of groups). **C** Mean CDR3 length of TCRβ repertoire shown in IgAN (red), non-IgAN (green), and healthy (blue) subjects. Differences in means CDR3 length were examined by the Kruskal–Wallis test (among three groups) and the Wilcoxon rank sum test (between any two of groups). **D** Gene usages of *TRBV5-4*, *TRBV6-4*, *TRBV12-1*, *TRBV16*, and *TRBV21-1* shown in IgAN (red), non-IgAN (green), and healthy (blue) subjects. Differences in gene usage were calculated by the Wilcoxon rank sum test. *P*-value < 0.05 was considered statistically significant and is denoted by “*”

**Table 1:** Baseline characteristics of IgAN patients, non-IgAN patients, and healthy controls

	**IgAN** (*n* = 8)	**Non-IgAN** (*n* = 25)	**Healthy** (*n* = 10)	***P*-value**	**Adjusted** *p*-value^*^
**Sex (female/male)**	3/5	8/17	5/5	0.6093	1.0000
**Age (mean)**	47.5	58.5	54.9	0.0945	0.3781
**Diabetic nephropathy**	-	10	-	-	-
**Hypertensive nephropathy**	-	15	-	-	-
**eGFR (mean, mL/min/1.73m**^**2**^**)**	55.2	51.2	98.8	**0.000166**	**0.000663**
**UPCR (mean, g/day)**	1.09	1.45	0.07	0.1136	0.4546

*Abbreviation*: *eGFR* estimated glomerular filtration rate, *UPCR* urine protein/creatinine ratio

^*^P-value was adjusted by Bonferroni correction

For TCRβ repertoire diversity, no significant difference between IgAN, non-IgAN, and healthy group was observed. However, we noted that the distribution of the diversity index in IgAN patients was slightly lower than that in non-IgAN patients and in healthy individuals (Fig. 1B). The analysis of TCRβ repertoire space indicated that IgAN patients tended to exhibit higher proportion of hyperexpanded clone size than non-IgAN patients and healthy individuals, suggesting a clonal expansion within TCRβ repertoire in IgAN patients (Figure S1). These results are consistent with previous findings that observed a non-significant but mild decrease in the Shannon index of peripheral TCRβ repertoire in IgAN patients relative to healthy individuals [7].

By comparing CDR3 length distribution of TCRβ repertoire, we further noticed a tendency toward a higher usage of short CDR3 lengths in non-IgAN patients (Figure S2A). Indeed, the analysis of mean CDR3 length revealed that non-IgAN patients had lower means of CDR3 lengths than IgAN patients (Fig. 1C). Our results highlighted a potential divergence in CDR3 lengths of TCRβ repertoire between IgAN and non-IgAN patients. By contrast, no difference in CDR3 length distribution and usage between IgAN patients and healthy individuals was found. Similar result was reported by a previous study [6]. However, another large cohort study showed a significant bias toward shorter CDR3 lengths in IgAN patients compared to healthy controls, nicely suggesting a significant role of CDR3 length distribution between IgAN and healthy subjects [7].

In TCR gene usage analysis, although no significant difference in overall TRBV and TRBJ usages between IgAN, non-IgAN, and healthy group was observed (Figure S2B and S2C), we found that *TRBV5-4*, *TRBV6-4*, *TRBV12-1*, *TRBV16*, and *TRBV21-1* exhibited statistical differences in gene usage between IgAN, non-IgAN, and healthy subjects. (Figure S3). Importantly, the usages of *TRBV6-4*, *TRBV12-1*, and *TRBV21-1* in IgAN patients were significantly higher than those in healthy individuals and/or in non-IgAN patients (Fig. 1D). Furthermore, the higher usage of *TRBV6-4* in IgAN patients implied that mucosal-associated invariant T (MAIT) cells, which are characterized by invariant TCRα associated with Vβ2 and Vβ13 chains (*TRBV6* and *TRBV20*) and have been known to be involved in glomerulonephritis, may participate in the pathogenesis and/or development of IgAN [8, 9].

In summary, we performed TCR repertoire sequencing for IgAN patients, non-IgAN patients, and healthy controls. Our results regarding TCRβ repertoire diversity and CDR3 length distribution in IgAN patients relative to healthy individuals are consistent with existing findings from previous studies. Importantly, we identified the difference in CDR3 length distribution between IgAN and non-IgAN patients and reported a higher usage of MAIT-associated gene *TRBV6-4* in IgAN patients. Additionally, we recognize the limitations of our study as follows: (1) The sample size in IgAN group was relatively small that might reduce the statistical power and increase the variation of the analysis. (2) Other types of non-IgA glomerulonephritis, such as membranous nephropathy and lupus nephritis, were not included for the comparison to IgAN. Nonetheless, on the basis of these findings, the characteristics of TCRβ repertoire is suggested with potential to diagnose IgAN.

### Supplementary Information

**Additional file1 :** **Supplementary Methods. Table S1. **Baseline characteristics of all cohort subjects. **Figure S1. **Comparison of T-cell receptor beta-chain (TCRβ) repertoire space between IgAN, non-IgAN, and healthy group. **Figure S2**. Complementarity determining region (CDR3) length distribution and principal component analysis (PCA) for TRBV or TRBJ gene usage in IgAN, non-IgAN, and healthy group. **Figure S3**. Comparison of all TRBV or TRBJ gene usages between IgAN, non-IgAN, and healthy group.

## Data Availability

All data related to this article are shown or available upon request from the corresponding authors.

## Abbreviations

IgAN: Immunoglobulin A nephropathy
ESRD: End-stage renal disease
TCR: T-cell receptor
TCRβ: T-cell receptor beta-chain
TRB: T-cell receptor beta locus
CDR3: Complementarity determining region 3
MAIT: Mucosal-associated invariant T
