# Supplementary material for: Characterization of T-Cell receptor repertoire in immunoglobulin a nephropathy
Source: Biomark Res. 2024 Feb 12;12:23. doi: 10.1186/s40364-024-00572-2 (PMC10860214; doi:10.1186/s40364-024-00572-2)
Supplement: Supplementary file 1 — Additional file1 : Supplementary Methods. Table S1. Baseline characteristics of all cohort subjects. Figure S1. Comparison of T-cell receptor beta-chain (TCRβ) repertoire space between IgAN, non-IgAN, and healthy group. Figure S2. Complementarity determining region (CDR3) length distribution and principal component analysis (PCA) for TRBV or TRBJ gene usage in IgAN, non-IgAN, and healthy group. Figure S3. Comparison of all TRBV or TRBJ gene usages between IgAN, non-IgAN, and healthy group. [file 40364_2024_572_MOESM1_ESM.pdf]

Supplementary Methods

Study population

The study was approved by Taipei Medical University-Joint Institutional Review Board and all subjects signed the informed consent forms (IRB number: N201704064). We recruited 8 IgAN patients, 25 non-IgAN patients, and 10 healthy controls from Taipei Medical University Hospital. IgAN patients who were diagnosed by renal biopsy were included. Non-IgAN patients were recruited from patients diagnosed with hypertensive nephropathy or diabetic nephropathy. Subjects with eGFR >60 and without proteinuria were included as healthy controls. Exclusion criteria were: (1) ages > 75 years; (2) history of cancers or autoimmune diseases; (3) use of immunosuppressive drugs 1 month before sampling. We also collected the subject's serum creatinine, eGFR value, and urine protein/creatinine ratio to assess renal function. Especially, the mesangial hypercellularity (M), endocapillary hypercellularity (E), segmental glomerulosclerosis (S), and tubular atrophy/interstitial fibrosis (T) score (MEST) was used to classify the severity of IgAN based on histopathology [1]. Finally, the peripheral blood was drawn from every subject.

TCR library preparation and sequencing

After blood drawing, we isolated peripheral blood mononuclear cells (PBMCs) from the peripheral whole blood using Histopaque-1077 (Sigma-Aldrich, MO, USA). PBMCs were then stored with dimethyl sulfoxide (Sigma-Aldrich, MO, USA) and 50%-FBS RPMI medium (Thermo Fisher Scientific, MA, USA) in liquid nitrogen. After collecting a batch of PBMC samples, RNA was subsequently extracted from PBMCs using AllPrep DNA/RNA Mini kit (Qiagen, Netherlands). Before library preparation, RNA quality was evaluated by Qsep 100 (Bioptic, Taiwan). Samples with RNA quality number (RQN) > 4 were then subjected for TCR library preparation. TCR enrichment was based on 5' RACE technology with customized primers. We used 1.5% Agarose Dye-Free DNA gel cassettes (Sage Science, MA, USA) to select TCR fragments which ranged from 600 to 900 bps. All fragments from PCR and size selection were purified using Agencourt AMPure XP (Beckman Coulter, CA, USA). Each DNA fragment was ligated with sequencing adaptors and indices by Nextera XT Index Kit (Illumina, CA, USA). Finally, TCR libraries were pooled together and sequenced on the Miseq platform (Illumina, CA, USA).

TCR sequencing data preprocessing

Raw sequencing data were processed through a customized pipeline which was constructed by Cutadapt software (version 3.7), MIGEC software (version 1.2.9), and MiXCR software (version 3.0.13). We first trimmed adaptor sequences and removed low-quality reads using Cutadapt. We next conducted unique molecular identifier (UMI) calling and PCR error correction using MIGEC. After correction, consensus UMI reads were aligned to V and J segments of T-cell receptor beta locus (TRB), and CDR3 sequences were determined from aligned reads as TCRβ clonotypes. Finally, identical CDR3 sequences were determined as unique clonotypes. Given the frequency of unique TCRβ

clonotypes, we subsequently compared characteristics of TCRβ repertoire between different clinical groups by downstream analyses.

TCR repertoire diversity

TCR repertoire diversity was estimated by the Shannon index. A random subsample of 8764 clones was generated to calculate the diversity index, which was then used to determine the mean of 100 repeats for each clinical sample. Formulas of the diversity index is as follows:

$$Shannon\ index\ (H') = -\sum_{i=1}^{n} p_i \times log_2(p_i)$$

where n indicates the total number of TCR clonotypes and p_i represents the frequency of the ith clonotype of TCR repertoire.

Distribution and mean of CDR3 lengths

In the data preprocessing, we removed non-functional CDR3 sequences to retain functional CDR3 sequences for analysis. CDR3 length was determined by the number of amino acids of unique CDR3 sequences. We analyzed both distribution and mean of CDR3 lengths. A total of 8764 clones were randomly subsampled to calculate the distribution and the mean of CDR3 lengths, which were subsequently utilized to determine means of 100 repeats for each clinical sample.

Usage of TRBV and TRBJ genes

The TRBV/TRBJ gene usage was determined by the frequency of TRBV/TRBJ gene used by TCR clonotypes in a TCRβ repertoire. A random subsample of 8764 clones was generated to calculate the TRBV and TRBJ gene usages, which were then used to determine the means of 100 repeats for each clinical sample. TRBJ2-2P as a pseudogene was removed from the analysis.

Statistical analysis

We used immunarch package (version 0.7.0) in R (version 4.1.2) for all the analysis. For comparison of two groups, the Wilcoxon rank-sum test was used. For comparison of three groups, we used the Kruskal-Wallis test. Categorical variables were examined by chi-squared test.

Table S1. Baseline characteristics of all cohort subjects.

Sample	Group	Age	Sex	MEST/Subgroup	ISx cessation†	Cr	eGFR	Uprotein	UPCR
S001	IgAN	58	Male	M1E0S1T2	15 months	3.3	21	60.2	0.79
S006	IgAN	30	Male	M0E0S1T0	None	1.1	86	70.7	0.68
S019	IgAN	63	Male	M1E0S1T2	None	1.4	55	190.2	2.38
S036	IgAN	40	Female	N/A	None	1.4	45	8.2	0.18
S052	IgAN	61	Female	M1E0S1T0	None	0.8	78	N/A	N/A
S053	IgAN	51	Female	M1E0S0T1	None	1.3	46	172.2	1.42
S054	IgAN	49	Male	M0E1S1T1	25 months	N/A	N/A	N/A	N/A
S106	IgAN	28	Male	M1E0S1T2	5 months	N/A	N/A	N/A	N/A
S007	Non-IgAN	69	Male	Hypertensive nephropathy	N/A	1.1	71	N/A	N/A
S010	Non-IgAN	54	Male	Hypertensive nephropathy	N/A	1.2	68	16.2	0.06
S011	Non-IgAN	68	Male	Diabetic nephropathy	N/A	1.6	68	N/A	N/A
S020	Non-IgAN	54	Male	Diabetic nephropathy	N/A	2.5	29	421	3.24
S022	Non-IgAN	65	Female	Diabetic nephropathy	N/A	0.9	68	25.6	0.11
S038	Non-IgAN	64	Male	Hypertensive nephropathy	N/A	1.8	41	N/A	N/A
S041	Non-IgAN	69	Male	Hypertensive nephropathy	N/A	1.7	43	11.4	0.11
S044	Non-IgAN	46	Male	Hypertensive nephropathy	N/A	1.9	41	16.7	0.11
S056	Non-IgAN	56	Female	Diabetic nephropathy	N/A	1.7	33	152.6	8.16
S061	Non-IgAN	61	Female	Hypertensive nephropathy	N/A	0.9	68	4.5	0.08
S062	Non-IgAN	35	Male	Diabetic nephropathy	N/A	2.3	35	247.4	1.56
S063	Non-IgAN	66	Male	Diabetic nephropathy	N/A	2.1	34	151.6	0.73

S065	Non-IgAN	69	Male	Hypertensive nephropathy	N/A	1.6	46	N/A	N/A
S072	Non-IgAN	45	Male	Hypertensive nephropathy	N/A	3.7	19	75.9	0.43
S073	Non-IgAN	61	Female	Diabetic nephropathy	N/A	1.8	31	246	3.25
S077	Non-IgAN	62	Female	Hypertensive nephropathy	N/A	N/A	N/A	N/A	N/A
S079	Non-IgAN	67	Male	Hypertensive nephropathy	N/A	1.4	54	N/A	N/A
S090	Non-IgAN	65	Female	Diabetic nephropathy	N/A	0.8	77	N/A	N/A
S095	Non-IgAN	53	Male	Hypertensive nephropathy	N/A	1.4	56	11.9	0.06
S096	Non-IgAN	64	Male	Hypertensive nephropathy	N/A	1.1	71	N/A	N/A
S097	Non-IgAN	37	Male	Hypertensive nephropathy	N/A	1.2	73	388.9	2.37
S102	Non-IgAN	49	Male	Diabetic nephropathy	N/A	N/A	N/A	N/A	N/A
S103	Non-IgAN	60	Female	Hypertensive nephropathy	N/A	N/A	N/A	N/A	N/A
S104	Non-IgAN	59	Male	Hypertensive nephropathy	N/A	1.1	73	14.3	0.06
S108	Non-IgAN	64	Female	Diabetic nephropathy	N/A	1.9	28	N/A	N/A
S028	Healthy control	67	Female	N/A	N/A	0.4	171	N/A	N/A
S035	Healthy control	60	Male	N/A	N/A	0.8	106	N/A	N/A
S040	Healthy control	59	Female	N/A	N/A	0.7	92	N/A	N/A
S047	Healthy control	70	Male	N/A	N/A	1	79	N/A	N/A
S048	Healthy control	69	Male	N/A	N/A	1	79	N/A	N/A
S055	Healthy control	68	Female	N/A	N/A	1	59	N/A	N/A
S088	Healthy control	23	Male	N/A	N/A	1	99	18.8	0.04
S092	Healthy control	37	Female	N/A	N/A	0.6	121	8.7	0.11
S094	Healthy control	64	Female	N/A	N/A	0.8	77	7.7	0.08

S099	Healthy control	32	Male	N/A	N/A	0.9	105	N/A	N/A

† The time interval between the cessation of immunosuppressant administration and the collection of blood samples.

Abbreviation: ISx, immunosuppressant; Cr, Creatinine (mg/dl); eGFR, estimated glomerular filtration rate (mL/min/1.73m2); Uprotein, urine protein (mg/dl); UPCR, urine protein/creatinine ratio (g).

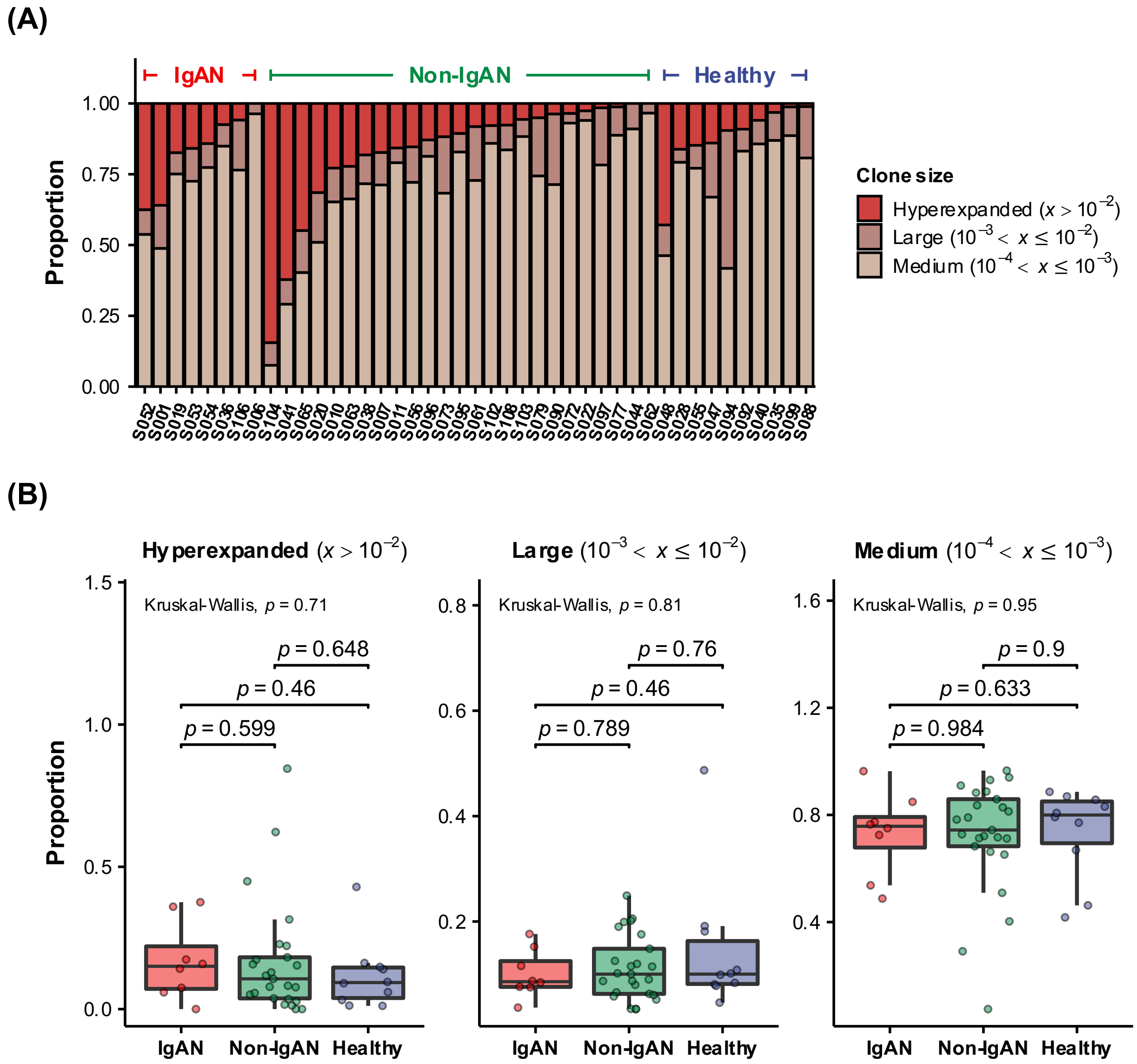

Figure S1. Comparison of T-cell receptor beta-chain (TCRβ) repertoire space between IgAN, non-IgAN, and healthy group. (**A**) Distribution of clone sizes of TCRβ repertoire space in IgAN (red), non-IgAN (green), and healthy (blue) subjects. Each clonotype was categorized based on its frequency (x) into hyperexpanded ($x > 10^{-2}$), large ($10^{-2} \ge x > 10^{-3}$), or medium ($10^{-3} \ge x > 10^{-4}$) clone size. The frequencies of clonotypes of each clone size was added as clone size proportion. (**B**) Proportion of medium (left panel), large (middle panel), and hyperexpanded (right panel) clone size in subjects of the three groups. Differences in clone size proportion were calculated by the Kruskal-Wallis test (among three groups) and the Wilcoxon rank sum test (between any two of groups).

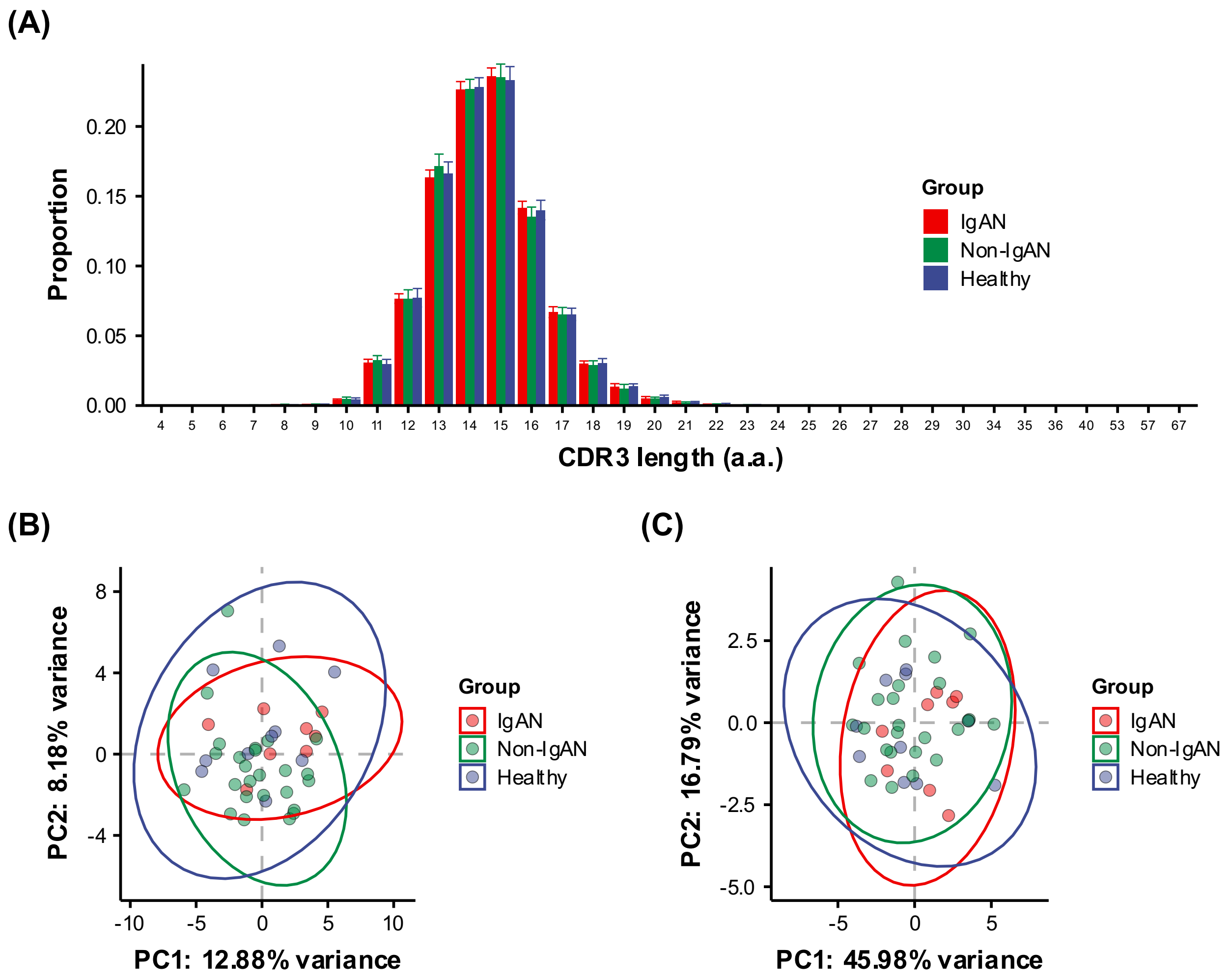

Figure S2. Complementarity determining region (CDR3) length distribution and principal component analysis (PCA) for TRBV or TRBJ gene usage in IgAN, non-IgAN, and healthy group. (**A**) Distribution of CDR3 amino acid lengths in IgAN (red), non-IgAN (green), and healthy (blue) groups. The height and error bar represent the mean and the standard deviation of CDR3 length proportions, respectively. (**B**) PCA for the usage of TRBV genes in IgAN (red), non-IgAN (green), and healthy (blue) subjects. (**C**) PCA for the TRBJ gene usage in IgAN (red), non-IgAN (green), and healthy (blue) subjects. The ellipse represents the 95% confidence interval for the IgAN (red), non-IgAN (green), and healthy (blue) group.

(A)

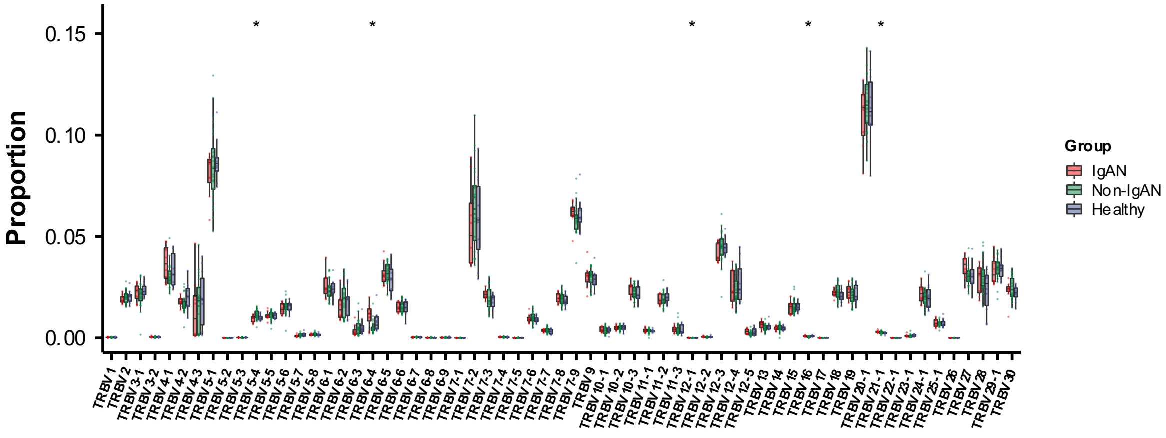

(B)

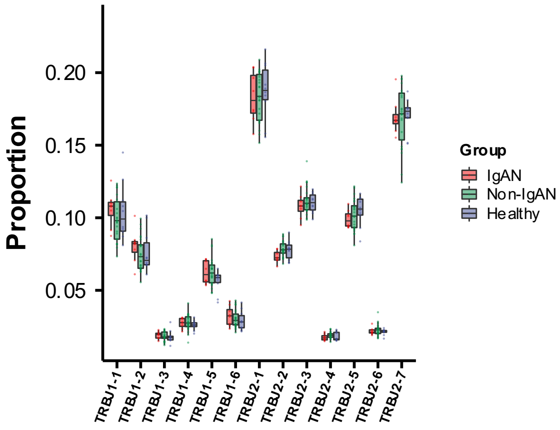

Figure S3. Comparison of all TRBV or TRBJ gene usages between IgAN, non-IgAN, and healthy group. Gene usages of all the observed TRBV (**A**) and TRBJ (**B**) genes in IgAN (red), non-IgAN (green), and healthy (blue) subjects. Differences in gene usage were calculated by the Kruskal-Wallis test. *P*-value < 0.05 was considered statistically significant and is denoted by "*".

References

1. Cattran DC, Coppo R, Cook HT, Feehally J, Roberts IS, Troyanov S, et al. The Oxford classification of IgA nephropathy: rationale, clinicopathological correlations, and classification. Kidney international. 2009;76(5):534-45.
